# Supplementary material for: Managing incontinence in low-and middle income-countries: A qualitative case study from Pakistan
Source: PLoS One. 2022 Jul 15;17(7):e0271617. doi: 10.1371/journal.pone.0271617 (PMC9286225; doi:10.1371/journal.pone.0271617)

PLOS ONE

Managing incontinence in low-and middle income-countries: A qualitative case study from Pakistan

--Manuscript Draft--

Manuscript Number:	PONE-D-22-04296
Article Type:	Research Article
Full Title:	Managing incontinence in low-and middle income-countries: A qualitative case study from Pakistan
Short Title:	Managing incontinence in low-and middle income-countries: A qualitative case study from Pakistan
Corresponding Author:	Sian White<br>London School of Hygiene and Tropical Medicine<br>London, UNITED KINGDOM
Keywords:	Incontinence; Pakistan; disability; qualitative methods; caregivers
Abstract:	Background: Incontinence is a complex health and social issue, which involves the involuntary loss of urine or faeces or both. Individuals with disabilities are particularly vulnerable to incontinence. The management of incontinence has largely been overlooked in low and middle-income settings (LMICs). This study aimed to explore the incontinence management strategies employed by disabled people with severe incontinence and their caregivers in Sindh Province, Pakistan.<br>Methods: Incontinence management was explored through in-depth interviews with people with incontinence (PWI) and their caregivers, photovoice, and a market survey and product attribute assessment. Data was analysed thematically through inductive coding and evaluated against existing disability and caregiver frameworks.<br>Results: Incontinence management affected all aspects of daily life for PWI and caregivers. Effective management of incontinence was prioritised because caregivers viewed it to be part of their familial duty and a requirement for the household to remain pure in the eyes of God. Coping strategies included strict adherence to routines, reducing food and drink intake, creative uses of locally available natural resources, and a heavy reliance on soap and water for maintaining hygiene. Products such as adult diapers were largely unavailable, costly and were not deemed suitable for regular use. There were no social or medical interventions in the region to support incontinence management. The main impacts of incontinence on the household were social isolation, stigma, role shifts within the family, the development of physical ailments among caregivers, and decreased income.<br>Conclusion: The complex health, psychological, social, economic, and cultural impacts of incontinence are exacerbated in LMICs due a lack of recognition of the condition, the absence of social or medical interventions and limited access to basic WASH infrastructure, and assistive devices or products. Appropriate solutions need to be developed in partnership with PWI and caregivers and need to be contextualised, affordable and sustainable.
Order of Authors:	Zara Ansari
	Sian White
Additional Information:	
Question	Response
Financial Disclosure<br><br>Enter a financial disclosure statement that describes the sources of funding for the work included in this submission. Review the submission guidelines for detailed requirements. View published research articles from *PLOS ONE* for specific	The authors received no specific grant funding for this work. Travel and publication costs were supported by Norwegian Church Aid and the London School of Hygiene and Tropical Medicine. The funders had no role in study design, data collection and analysis, decision to publish, or preparation of the manuscript.

Powered by Editorial Manager® and ProduXion Manager® from Aries Systems Corporation

examples.<br><br>This statement is required for submission and **will appear in the published article** if the submission is accepted. Please make sure it is accurate.<br><br>**Unfunded studies**<br>Enter: *The author(s) received no specific funding for this work.*<br><br>**Funded studies**<br>Enter a statement with the following details:<br>• Initials of the authors who received each award<br>• Grant numbers awarded to each author<br>• The full name of each funder<br>• URL of each funder website<br>• Did the sponsors or funders play any role in the study design, data collection and analysis, decision to publish, or preparation of the manuscript?<br>• **NO** - Include this sentence at the end of your statement: *The funders had no role in study design, data collection and analysis, decision to publish, or preparation of the manuscript.*<br>• **YES** - Specify the role(s) played.<br><br>* typeset	
**Competing Interests**<br><br>Use the instructions below to enter a competing interest statement for this submission. On behalf of all authors, disclose any competing interests that could be perceived to bias this work—acknowledging all financial support and any other relevant financial or non-financial competing interests.<br><br>This statement is required for submission and **will appear in the published article** if the submission is accepted. Please make sure it is accurate and that any funding sources listed in your Funding Information later in the submission form are also declared in your Financial Disclosure statement.	The authors have declared that no competing interests exist.

Powered by Editorial Manager® and ProduXion Manager® from Aries Systems Corporation

View published research articles from *PLOS ONE* for specific examples.<br><br>**NO authors have competing interests**<br><br>Enter: *The authors have declared that no competing interests exist.*<br><br>**Authors with competing interests**<br><br>Enter competing interest details beginning with this statement:<br><br>*I have read the journal's policy and the authors of this manuscript have the following competing interests: [insert competing interests here]*<br><br>* typeset	
**Ethics Statement**<br><br>Enter an ethics statement for this submission. This statement is required if the study involved:<br><br>• Human participants<br>• Human specimens or tissue<br>• Vertebrate animals or cephalopods<br>• Vertebrate embryos or tissues<br>• Field research<br><br>Write "N/A" if the submission does not require an ethics statement.<br><br>General guidance is provided below. Consult the submission guidelines for detailed instructions. **Make sure that all information entered here is included in the Methods section of the manuscript.**	The study received ethical approval from the London School of Hygiene and Tropical Medicine (ID:13595) and the Interactive Research and Development's Independent Institutional Review Board in Pakistan (ID: IRD_IRB_2017-05-002). All participants and caregivers provided informed written consent. A particularly detailed process of informed consent was done with photo voice participants. This involved PWI being able to select how their images could be used and shared and whether they wished their name or a pseudonym to be used.

Powered by Editorial Manager® and ProduXion Manager® from Aries Systems Corporation

**Format for specific study types**<br><br>**Human Subject Research (involving human participants and/or tissue)**<br>• Give the name of the institutional review board or ethics committee that approved the study<br>• Include the approval number and/or a statement indicating approval of this research<br>• Indicate the form of consent obtained (written/oral) or the reason that consent was not obtained (e.g. the data were analyzed anonymously)<br><br>**Animal Research (involving vertebrate animals, embryos or tissues)**<br>• Provide the name of the Institutional Animal Care and Use Committee (IACUC) or other relevant ethics board that reviewed the study protocol, and indicate whether they approved this research or granted a formal waiver of ethical approval<br>• Include an approval number if one was obtained<br>• If the study involved *non-human primates*, add *additional details* about animal welfare and steps taken to ameliorate suffering<br>• If anesthesia, euthanasia, or any kind of animal sacrifice is part of the study, include briefly which substances and/or methods were applied<br><br>**Field Research**<br><br>Include the following details if this study involves the collection of plant, animal, or other materials from a natural setting:<br>• Field permit number<br>• Name of the institution or relevant body that granted permission	
**Data Availability**<br><br>Authors are required to make all data underlying the findings described fully available, without restriction, and from the time of publication. PLOS allows rare exceptions to address legal and ethical concerns. See the PLOS Data Policy and FAQ for detailed information.	No - some restrictions will apply

A Data Availability Statement describing where the data can be found is required at submission. Your answers to this question constitute the Data Availability Statement and **will be published in the article**, if accepted.<br><br>**Important:** Stating 'data available on request from the author' is not sufficient. If your data are only available upon request, select 'No' for the first question and explain your exceptional situation in the text box.<br><br>Do the authors confirm that all data underlying the findings described in their manuscript are fully available without restriction?	
**Describe where the data may be found in full sentences. If you are copying our sample text, replace any instances of XXX with the appropriate details.**<br><br>• If the data are **held or will be held in a public repository**, include URLs, accession numbers or DOIs. If this information will only be available after acceptance, indicate this by ticking the box below. For example: *All XXX files are available from the XXX database (accession number(s) XXX, XXX.*).<br>• If the data are all contained **within the manuscript and/or Supporting Information files**, enter the following: *All relevant data are within the manuscript and its Supporting Information files.*<br>• If neither of these applies but you are able to provide **details of access elsewhere**, with or without limitations, please do so. For example:<br><br>*Data cannot be shared publicly because of [XXX]. Data are available from the XXX Institutional Data Access / Ethics Committee (contact via XXX) for researchers who meet the criteria for access to confidential data.*<br><br>*The data underlying the results presented in the study are available from (include the name of the third party*	Interview transcripts cannot be shared publicly because of confidentiality and risks of deductive disclosure. Data are available from the authors for researchers who meet the criteria for access to confidential data.

and contact information or URL).<br>• This text is appropriate if the data are owned by a third party and authors do not have permission to share the data.<br><br>* typeset	
Additional data availability information:	

Powered by Editorial Manager® and ProduXion Manager® from Aries Systems Corporation

Manuscript
Click here to access/download;Manuscript;Incontinence Paper_PO.docx

Managing incontinence in low-and middle income-countries: A qualitative case study from Pakistan

Zara Ansari[1], Sian White[2*]

[1]Currently with Acasus, Gizri Road, Block 9 Clifton, Karachi, Pakistan

[2] Department of Disease Control, London School of Hygiene and Tropical Medicine, Keppel Street, London, UK.

Corresponding author:

Sian.white@lshtm.ac.uk

Contributions:

ZA – Data curation, formal analysis, investigation, methodology, writing – original draft preparation. SW – Conceptualisation, funding acquisition, supervision, methodology, writing – review and editing.

Acknowledgements:

We would like to thank our research participants for their time and willingness to speak openly about living with incontinence. This research was made possible through the support of Benedicte Hafskjold, Åshild Skare and Corinna Schüttler-Kvarme at Norwegian Church Aid's (NCA) headquarters and Sumera Izhar, Zohaib Hassan, Shah Khalid from NCA in Pakistan who supported with data collection, logistics and access to the study sites. Travel and logistics costs associated with this research were covered by NCA and the London School of Hygiene and Tropical Medicine. Costs associated with the publication of this research were provided by the Environmental Health Group at the London School of Hygiene and Tropical Medicine.

Abstract

Background: Incontinence is a complex health and social issue, which involves the involuntary loss of urine or faeces or both. Individuals with disabilities are particularly vulnerable to incontinence. The management of incontinence has largely been overlooked in low and middle-income settings (LMICs). This study aimed to explore the incontinence management strategies employed by disabled people with severe incontinence and their caregivers in Sindh Province, Pakistan.

Methods: Incontinence management was explored through in-depth interviews with people with incontinence (PWI) and their caregivers, photovoice, and a market survey and product attribute assessment. Data was analysed thematically through inductive coding and evaluated against existing disability and caregiver frameworks.

Results: Incontinence management affected all aspects of daily life for PWI and caregivers. Effective management of incontinence was prioritised because caregivers viewed it to be part of their familial duty and a requirement for the household to remain pure in the eyes of God. Coping strategies included strict adherence to routines, reducing food and drink intake, creative uses of locally available natural resources, and a heavy reliance on soap and water for maintaining hygiene. Products such as adult diapers were largely unavailable, costly and were not deemed suitable for regular use. There were no social or medical interventions in the region to support incontinence management. The main impacts of incontinence on the household were social isolation, stigma, role shifts within the family, the development of physical ailments among caregivers, and decreased income.

Conclusion: The complex health, psychological, social, economic, and cultural impacts of incontinence are exacerbated in LMICs due a lack of recognition of the condition, the absence of social or medical interventions and limited access to basic WASH infrastructure, and assistive devices or products. Appropriate solutions need to be developed in partnership with PWI and caregivers and need to be contextualised, affordable and sustainable.

Introduction:

Urinary and faecal incontinence is a complex public health issue with a range of social and health consequences. Urinary incontinence (UI) is defined as any involuntary loss of urine (1) whereas faecal incontinence (FI) is the inability to control bowel movements, resulting in the involuntary passage or leakage of stools (2). Incontinence most commonly effects older people, multiparous women, children and people with physical or cognitive impairments (3). The global burden of incontinence is difficult to estimate. Most epidemiological studies are from high income countries, (4–6) where the prevalence of UI is estimated to be 28% in women, and 10% in men.

Incontinence impacts the physical and mental health status of the individual. Incontinence is associated with urinary tract infections, higher exposure to fecal oral pathogens, increased number of bacterial and fungal skin infections and higher prevalence of depression and anxiety (4,7,8). The health-related quality of life (HRQoL) impact of incontinence is similar to that observed with other chronic medical conditions like osteoarthritis, chronic obstructive pulmonary disease, and stroke.(4)

The shame and public stigma of incontinence affects people's treatment seeking behaviour (9–13) with most not seeking treatment because of embarrassment or low expectations of treatment outcomes. Incontinence can impact a person's ability to fully participate in society, for example in the workplace it can reduce working hours and have negative impacts on concentration and performance of physical activities (5)

Research from high income countries has shown that incontinence management places significant economic burdens on both the patient and the health system (6). In 2000, the total cost of managing incontinence in the US was estimated to be USD19.5 billion (14).

In high income countries, a wide variety of products and support services are available to manage incontinence. These products are available in pharmacies and supermarkets or even online. These include but are not limited to disposable diapers or inserts, reusable pull up pants, disposable catheters and disposable mattress protectors (6). In high income settings water and soap are typically readily available – both of which are key in effectively managing incontinence.

While little is known about how incontinence is managed in low-and middle-income countries (LMICs), a recent study in Vanuatu highlighted a range of barriers including lack of incontinence products or assistive devices, social stigma towards the condition and isolation of people with incontinence (15).

This study was designed to understand the coping mechanisms employed by older people or people with disabilities and their families to manage incontinence in a LMIC setting. The study also explored the relative importance incontinence management in comparison to other issues of daily life for older people or people with disabilities.

Methods

Study site

This study was conducted in Sindh Province in Pakistan in July 2017, during the rainy season. According to the 2004 World Health Survey, the prevalence of disability in Pakistan is approximately 13% (16). Water, sanitation and hygiene (WASH) coverage varies substantially between urban and rural regions of Pakistan. In urban areas, basic sanitation coverage is estimated to be 76%, while in rural it is 50%. Similarly, in urban areas the majority of people (86%) have access to a basic handwashing facility with soap and water but in rural areas 54% of the population lack this. Only 35% of Pakistan's population have access to safely managed water (17). At the time of this study there was displacement due to flooding in several regions of Sindh.

Data Collection

The study employed a range of participatory qualitative methods including in-depth interviews with people with incontinence and their caregivers, photovoice, a market survey of incontinence products, and field note taking. All these methods are described in detail in Table 1.

Table 1: Detailed Description of Participatory Qualitative Methods

Description	Sample Size
**In-depth Interviews:** We developed an open-ended question guide that focused on the management of incontinence. These included questions on incontinence management, coping mechanisms (individual and of their carers), barriers, product use and access to water, sanitation and hygiene (WASH).	10 people with incontinence and their caregivers (5 of these participant-carer pairs lived in urban settings and 5 in rural settings). In each pair the person with incontinence was interviewed separately to the carer so that they both were able to speak freely about their experiences.
**Photovoice:** This aimed to understand the impact of incontinence within the larger context of issues faced by people with disabilities. Respondents were given a camera	4 people with disabilities (2 urban and 2 rural)

and asked to take total of 10 pictures, 5 representing the best part of their day and 5 representing the challenges they face in managing their incontinence. This was followed by asking the participants to rank the pictures from most important to least important. This followed a protocol piloted in previous work with people with disabilities (18)	
**Examination of physical artefacts:** A market survey was conducted to understand the availability of products used for incontinence management. We approached a range of local shops including local pharmacies, general stores and local *thelas* (pushcart vendors) and asked them whether they stocked products for incontinence management. During in-depth interviews, families were asked to bring out all items they were using in the management of incontinence. They were asked a series of questions about each item including how long it has been used for and it's positive and negative attributes. Products purchased in the local market were introduced and a discussion took place on their opinion regarding the products and their perceived cost and utility.	Conducted with the same 10 participant pairs that took part in in-depth interviews.
**Field Notes**: The lead researcher (ZA) took extensive field notes during her research. The notes were used to capture key observations about behaviors and coping mechanisms that did not come across in the other methods.	Notes written at the end of each day of data collection.

Sample and Recruitment

Any person with either faecal and/or urinary incontinence was eligible to be included in the study as a primary participant. Hereafter primary participants are referred to as participants with incontinence (PWI). PWI were excluded if their incontinence could be repaired through medical intervention such as women with fistulas or men with prostate issues.

Caregivers of primary participants were also included as 'Secondary participants' if they were actively involved in assisting to manage the PWI incontinence.

In the first phase of sampling, a local disability person's organization (DPO), health workers, women's groups and village leaders were provided with a definition of incontinence and asked to list any

households where they knew of someone that had this challenge. Due to lack of awareness about incontinence, this strategy was amended to also list individuals with cognitive and physical impairments. Families with a person meeting this revised criterion were then visited to see if they were eligible. People with more severe incontinence were intentionally selected to participate, however participants were also purposively sampled from the list to reflect a variation in age, urban-rural location, socioeconomic status and impairment type.

Data management and analysis

Information gathered from all methods was audio-recorded and then transcribed orthographically in Urdu, reproducing all spoken words and sounds including hesitations, cut-offs in speech, laughter and long pauses. All transcripts were imported into NVivo 10 and thematically analysed using a six-step analysis method (19). A coding structure was developed inductively based on the phrases expressed by the participants (20), particularly through the PhotoVoice exercise. The codes were reviewed and constant comparisons were done to develop emerging themes. Themes were illustrated based on the quotes from the study. The data was summarised and visualised using disability and caregiver frameworks (21,22).

Ethical Approval and Consent

The study received ethical approval from the London School of Hygiene and Tropical Medicine (ID:13595) and the Interactive Research and Development's Independent Institutional Review Board in Pakistan (ID: IRD_IRB_2017-05-002). All participants and caregivers provided informed written consent. A particularly detailed process of informed consent was done with photo voice participants. This involved PWI being able to select how their images could be used and shared and whether they wished their name or a pseudonym to be used.

Results

Characteristics of Sample

Table 2 describes the characteristics of the participants and caregivers included in this study. All of the PWI had a physical or cognitive impairment, with majority being male as we could not identify any other primary female participants. All participants were considered to have severe incontinence, and were

either unable to use a toilet independently or were reliant on assistive products (e.g. diapers). Of the 10 caregivers, nine were women and all were family members, the majority being wives or mothers of the disabled person. Seven other elderly PWI (without visible physical/cognitive disability) were asked to be part of the study, but all refused to participate. All of these individuals stated that they did not have incontinence, even after their caregivers confirmed otherwise. Photovoice was conducted with four additional participants, all with a physical disability.

Table 2: Description of interview participants according to their role as either a person with incontinence or a caregivers

	**Participants with Incontinence**	**Caregivers**
Number of participants	10	10
**Age**		
Age range, years	22-70	19-75
**Gender**		
Male	9	1
Female	1	9
**Geographic location of participants**		
Urban	5	5
Rural	5	5
**Nature of Disability** §		
**Physical Impairment**		
Paralysis	7	
Limb Impairment	4	
Cerebral Palsy	2	
Polio	1	
Stroke	1	

**Cognitive/Intellectual Impairments**		
Intellectual Impairment	3	

§Some participants had more than one impairment

Prioritization of incontinence management within daily life

PWI and caregivers reported that incontinence management was always a priority in their day to day lives. Three themes emerged through interviews and photovoice to explain this prioritization: the importance of routine, the carer's sense of responsibility and the need to be clean in the eyes of God.

"Roz mara ka mamol" - Routine:

PWI and caregivers emphasized that incontinence management affects many aspects of their daily routines. Following a structured daily routine made managing incontinence easier for both carers and PWI. During a typical day caregivers explained that they would normally get up before the PWI so that they could address their own needs. Once the PWI was awake the carers day was dominated by tasks related to incontinence management. This included bathing the PWI, cleaning their clothes, and washing sheets or bedding from the night before. Over the years, most careers had noticed patterns in the timing of the bowel and urinary movements of the PWI and adjusted routines to align with this:

> *"She usually has to go around 11 and has a schedule. She will then have to go again around 1 30 and will tell me to quickly come home from work to take her because she can't control it otherwise"* – Caregiver of a PWI, urban area.

Accordingly, most caregivers were also responsible for providing food and drink to the PWI at certain times of the day so that incontinence management could be more easily predicted. Most caregivers were able to describe their daily routine schedules in detail. This level of structure was also described as necessary because activities such as bathing often required the involvement of more than one person.

"Ye meri zimidari hai" – It is my responsibility

Within the Pakistani context, providing care to family members was recognized to be part of cultural and religious norms. All caregivers framed their incontinence management work within the discourse of familial duty and responsibility. Specifically, caregivers believed it was their responsibility to preserve

the dignity of the PWI they were taking care of. Effective management of incontinence was key to maintaining that dignity, as sitting in urine or faeces was considered humiliating. Even if the task caused embarrassment for the caregiver, their sense of responsibility trumped those feelings:

> *"Sometimes I feel ashamed /embarrassed but after all he is part of my family and it is my responsibility to take care of him."* – Caregiver of a PWI, rural area.

Even though familial duty drove caregiving behaviour, incontinence management was not always done willingly. One caregiver said she was praised for taking care of her mother-in-law by others in the community, but it was a responsibility she did not want and hoped that her mother-in-law would soon pass away, so that this responsibility would be lifted from her.

"Paak nahi hain" – Not clean for God

The interaction between incontinence and faith was another reason management incontinence management was prioritized. Religion was seen as a way of making sense of incontinence and disability. Islam is dominant religion in Pakistan and governs many aspects of behaviour and daily life. Most PWI mentioned that Allah gave them this condition and felt that they owed it to God to remain clean:

> *"We have to have faith in God. Otherwise, what can we do? Whatever God does, that is what will happen. We can't change that. We can be clean to pray to him."* – PWI, urban setting

The two Hindu families included in this study also felt that they needed to remain clean in order to practice their religion and emphasized that it was therefore essential to prioritize cleaning and incontinence management over other aspects of their daily life.

Social, health and economic impact of incontinence

Six themes emerged to explain the impact of incontinence on the PWI and their caregiver.

"Tanhayee" – Isolation and Stigma

The social impact of incontinence manifested itself as isolation and stigma. While the majority of PWI stated that they did not feel isolated from their family members, a few mentioned relationship changes. The participant below describes how his relationship with his son changed over time:

> *"I feel that they have backed off. They don't ask me if they can help me..., they keep drifting away from us [PWI and caregiver]… and never turn around and ask about their father"*- PWI, rural setting

The majority of PWI did report feeling isolated from their friends and community members, which resulted in loneliness. PWI and caregivers rarely left their homes and did not participate in most social or cultural events. PWI attributed their isolation to a lack of awareness about their condition and the smell associated with incontinence. This finding also emerged from the photovoice activity (see Figure 1).

Figure 1: "They feel like I am a dirty person and do not want to be near me. I can't sit with my friends, most of them have left me and made me feel even more lonely and sad" Photo and caption by a PWI, rural setting

"Hamesha Nigrani Karna Parta Hai" - Constant Watchfulness

A noteworthy social impact for caregivers was social isolation which was experienced and mentioned by all. Some caregivers would only leave the house for a maximum of two hours, provided they could find an alternative person to cater for the participant's needs during the time they were away.

> *"The wife is with me all day. She is sitting on the bed with me all day. Whenever I need anything, she is with me all the time. She doesn't sleep. She sleeps for 5 minutes, then I call her and she is with me again."* PWI, rural setting

Caregivers were concerned that any lapse in 'watching over' the participant could result in additional work (e.g. the need for additional bathing), negative health consequences (e.g. damage to skin as a result of being stationary and sitting in urinary or faecal matter) and lead to the PWI experiencing feelings of guilt or embarrassment because they were not able to manage their incontinence independently. As such the social impact, isolation and stigma associated with incontinence permeated beyond the PWI to affect the caregiver.

"Zimidari badalna"- Role change

Many participants reported that with the onset of incontinence, they experienced a shift in gender-related roles and responsibilities, and that power dynamics within familial relationships changed. Pakistani culture is a patriarchal system which places most social liberties and decision-making in the

hands of men, however many of our PWI were male and received care from a female family member. In such circumstances PWI and caregivers described a 'role reversal' that substantially affected their social lives, sense of identity and ran counter to cultural norms. For example, the incontinence management provided by female caregivers led to altered power structures, with female caregivers now making most household-level decisions while male PWI felt completely dependent on them. This role change led to embarrassment in both parties, primarily when it came to bathing. Most caregivers felt that they should never have to see their male family members in such vulnerable positions:

> *"I have to hold him like a child when he goes to the bathroom or showers. He is my husband and I should never have to have become this person in his life. No one taught me how to make these decisions."* – Caregiver of a PWI, rural setting

> *"I am her husband. I was the man of the house and now look at me. I am dependent on her to lift me and clean me. This is not how a man should be. There is no dignity in this. A wife is not meant to do this. I am always ashamed and do not want to bathe."* – PWI, rural setting

"Khana Rokna" – Reducing food intake

Reducing food and drink intake emerged as one of the most coping mechanisms to manage incontinence in this setting. This had obvious negative health consequences. For example, it was observed that the majority of PWI were thin and carers explained that they did not want to eat. In Interviews and through photovoice PWI explained that limiting food and drink intake was a way for them to prevent embarrassment and reduce the burden on their caregivers, as highlighted by a male photovoice participant in Figure 2.

Figure 2: "I do stop eating food, especially when there are guests in the house. When the guests come, I stop eating and drinking all water so that I don't go to the bathroom or get dirty in front of them. I have to do my best to not be dirty and smell then" – photo and caption by a PWI, rural setting

However, choices about consumption did vary across PWI. Two participants said they increased their water intake to 'balance out' their choice to eat very little, explaining that this was necessary prevent dehydration during the hottest months of the year (temperatures sometimes got up to 52°C during the field work).

"Peeth main dard hogaya hai" - Development of physical ailments

Caregivers frequently reported that they had developed physical ailments because of their caregiving role. The most reported ailment was back and shoulder pains, associated with the regular need to lift and move PWI to bathe and clean them. One male photovoice participant chose to depict the effects caregiving had on his aging mother (Figure 3).

Figure 3: She has been taking care of me for so long now, but she can't see properly and walks with her head near the ground. Her back is getting curved taking care of me. – Photo and caption by a PWI, rural setting

Caregivers of PWI with cognitive impairments, reported frequent physical ailments and injuries. Maintenance of incontinence was even more difficult because, as one care giver explained, they *"can't carry him like a normal child".* Caregivers for PWI with cognitive impairments also mentioned being injured by impulsive behaviour, primarily when trying to clean the PWI. Sleep deprivation and fatigue were common among caregivers. One of the male PWI's mentioned how his caregivers do not seek healthcare for their health concerns:

> *"Their body begins to hurt, and I do not know how to make it not hurt. I tell them to go to the doctor, but they refuse. I think that I am light, but you don't realize that the body is very heavy and it will have an impact on the people that take care of you."*– PWI, urban setting.

Most PWI had not visited a doctor and those who had did so because of their disability and did not ask about their incontinence, due to embarrassment about the condition. One participant that did speak to the doctor about incontinence, was not given any advice on effectively managing it, being told to simply "*learn to live with it".* Another participant who had spoken to a doctor about his incontinence was provided with a catheter, but he was not told how to use this or any of the health issues that may be associated with its use. PWI explained that they didn't know how to raise the issue of incontinence with health professionals as there is no word for incontinence in the Urdu language.

"Paise hi nahi hain" – No more money

Financial difficulties came up as a common theme from both PWI and their caregivers. Participants explained that many people in their region lived in poverty - "*we are poor people and have no more money*." However, they added that their disability and incontinence exacerbated their poverty. Several PWI, especially males, said that they were unable to work or lost their jobs because of their disability and consequently, their incontinence. Caregivers were also faced with a choice between working or full-time care. Some caregivers spoke about selling their livestock and wedding jewellery to gather enough resources to support incontinence care.

Products for managing incontinence

Market survey

Table 3 provides a summary of the products available identified through the market survey and their costs. The most commonly available item for managing incontinence was adult diapers, with one brand of diapers being more common as it was made locally. All brands of diapers cost over GBP 5 for a pack of 10 diapers. Most items were available only in urban areas. Disposable pads or inserts, disposable mattress protectors and reusable pull up pants were not available in urban or rural areas.

Table 3: Incontinence management products and their price in GBP

Product	Location	Cost†
**Adult Diapers (Pack of 10)**	Karachi, Hyderabad and Mirpur Khas	£ 5 – 10
**Catheter**	Karachi and Hyderabad	£20-30
**Plastic Bed Sheets**	Karachi and Hyderabad	£4-5
**Dignity Sheets**	Karachi	£5-8

†*Conversion rate: GBP £1 = Pak Rs.140*

Shop owners and pharmacists who were asked about products for managing incontinence were often confused by the question. After describing the condition, some knew of adult diapers, but information on products was limited. One pharmacist handed the researcher a box of condoms thinking that this may help with the condition. Others seemed uncomfortable with the request and declined to provide suggestions of relevant products.

"Pampers se sub kaam nahi chalta" - Diapers are not the complete solution

When the products identified through the market survey were shown PWI and caregivers, most mentioned having seen diapers before, even in the rural areas of Sindh. However, when asked about the products they had available for incontinence management none of the five rural households and only two of the five urban households had diapers available and all reported that their use was minimal. Participants explained that diapers could temporarily reduce stress related to incontinence:

> *"They are okay. I wouldn't have to worry for a little while…. I wouldn't have to be worried about consistently dirtying my clothes then."* – PWI, Urban setting.

The problems associated with diapers were seen to outweigh the benefits. All families mentioned that the materials used in diapers meant that they were uncomfortable to use in hot weather with PWI being likely to get heat rashes and blisters from wearing the diapers. Caregivers for individuals with cognitive impairments used diapers more frequently. However, caregivers felt that diapers were not suitably created for adults with disabilities:

> *"Even then, it was very difficult for her to keep a diaper on. She didn't want it, said it was uncomfortable. It never fits her properly and then it leaks, so what is the point of having it on if it does not help at all?* – Caregiver of an PWI, urban setting.

The cost of diapers also deterred participants from using the product:

> *"Each diaper was for Rs 100. If I had to urinate 5 times a day, I will need Rs 500 every day. I didn't ask for it again."* PWI, rural setting.

The possibility of creating diapers at home seemed more appealing to participants. Cloth diapers were seen as more comfortable and cheaper alternative for the participants.

"Saabun aur paani hi chahiye" – We only need Soap and Water

Soap and water were repeatedly identified by PWI and caregivers as the most important products for managing incontinence and managing associated smells. The majority of the PWI took three to four showers a day to maintain their hygiene and minimize smell, with caregivers estimating that they often had to collect four or five times as much water to support their needs as compared to neighbouring households. PWI who had ample water available reported being happier as their laundry and bathing needs could be met in a timely and convenient manner. In rural areas, physical barriers, such as the distance to water points, were cited as most problematic by some caregivers:

"It takes a lot of effort to collect water. I have to go collect it almost three times a day and I can never carry enough for my brother's baths. He never feels clean enough and then sits and cries about his smell." - Caregiver of a PWI, urban setting.

In urban areas, some caregivers spoke about the costs of procuring enough water, indicating that this became a substantial part of their household income. All PWI and caregivers explained that soap was key for smell management and general cleanliness. This was captured by one participant in the photovoice activity (see Figure 4).

Figure 4: "This makes me feel very happy and clean. I use it all the time to manage my smell. It cleans my clothes, and it cleans my hands and the most important thing is that it keeps me clean." – Photo and caption by a PWI, urban area.

The majority of PWI and caregivers reported always having access to soap at home. While frequent soap purchasing was recognized as a significant expense, it was worth the cost and some families took loans from local stores to be able to have soap available. Scented soap was considered preferably by PWI and caregivers but was often more expensive.

"Humari zamin ka samaan" - Use of local products for incontinence management

Rural participants described the use of straw and grass as a tool for waste management:

"We have it lying under the bed. When he urinates or defecates, the liquid and some material seeps down into the grass. It is easy to clean as we then use a rake and push it to the side into this pile. The pile is then used to fertilize our crops." - PWI, rural setting.

Rural participants reported using sand as an alternative to soap to clean a PWI hands and body prior to being rinsed with water. Sand or clay were also used to cover the fecal matter so that it dried out and became easier to dispose of. These methods were seen as cost-effective solutions to incontinence management in rural settings. However, caregivers did emphasize that they were only used when they could no longer afford to purchase soap and that sand did not cover up the smell on the participants.

Discussion and Recommendations

Our research is the first to explore the lived experiences of people with severe incontinence and their caregivers in a LMIC setting. The emergent themes of this study highlight the need for a holistic approach to the management of incontinence in these settings. Across rural and urban areas of Sindh Province, incontinence was a condition that was largely undiagnosed and rarely discussed. The management of severe incontinence was found to affect all aspects of the daily lives of PWI and their caregivers. In a LMIC setting such as Pakistan, incontinence management created economic hardships for affected households, health challenges, social isolation from the wider community and had negative effects on the dignity, self-esteem and identity of those affected.

Figure 5 summarises the experiences of PWI within our study according to the International Classification of Functioning, Disability and Health (ICF) framework (21). Figure 6 summarises the experiences of caregivers for PWI according to the Organizing Framework for Caregiver Interventions (22). Both diagrams illustrate that present challenges with managing incontinence can best be addressed through targeted interventions that make it easier for PWI and caregivers to mitigate socio-economic and environmental barriers.

Figure 5: The International Classification of Functioning, Disability and Health (ICF) Framework (21) *adapted to summarise the experiences of people with severe incontinence in LMICs, based on our research*

Figure 6: The Organising Framework for Caregiver Interventions (22) adapted to summarise the experiences of caregivers for people with severe incontinence in LMICs, based on our research

Our findings are consistent with research documenting experiences of incontinence in high income settings (23–25). However, challenges related to managing incontinence appear to be exacerbated in LMICs due to factors in the socio-economic and physical environment. For example, in many high-income county settings there are professional care services, assistive products and devices, social protection schemes, health systems and formal or community-based support programmes which can help mitigate the costs and physical and mental toll of caring for a person with severe incontinence (23,26–29). While access and use of these services may still vary in high income settings, none of these support mechanisms were available to PWI or caregivers in our study. As such the financial costs and full

caring burden were borne by families and tended to compound existing levels of poverty, making such families particularly vulnerable.

Our findings are also consistent with the limited research that exists on incontinence in other LMICs. For example, research in Vanuatu, also found that there was no term for incontinence in the local language and that the condition was rarely discussed with medical professionals (15). They identified that incontinence products or assistive devises were unavailable or too expensive for regular use, that caregivers often developed physical ailments through caregiving work, and that PWI often reduced food and liquid intake as a key mitigation strategy. The research in Vanuatu suggested that a key mitigation strategy would be to improve access to conveniently located latrines, soap and water for families with a PWI and in public spaces. While this is an important step, our research indicates that for some people with severe incontinence we need to look at solutions beyond latrines. Recognition that toilet use may not be achievable for all is an important consideration given the framing of Sustainable Development Goal 6 (30) which aspires to provide sanitation for all. For our study participants, who had limited or no control over their bowel movements or loss of urine, regular bathing with soap and water and other mitigation techniques were more relevant. The increased water requirements needed by our study participants was consistent with findings from a study in Malawi which also found that households with a PWI needed five times as much water(18).

Below we discuss five domains of potential interventions that could be implemented by government and non-government organisations (NGOs) to make incontinence management easier in LMICs:

- Product-based interventions: This may include the local production of acceptable and reusable incontinence products; subsidies or vouchers for purchasing incontinence products (including soap); or the distribution of incontinence management kits which may include products such as scented soap, laundry detergent, cleaning products, plastic bed sheets, and dark-coloured clothing (so that incontinence stains are not easily visible). The sustainability of these types of product-based initiatives should be considered from the outset.
- Infrastructural interventions: Interventions in this category relate to how the home environment can be modified to facilitate easier incontinence management and may include the promotion of accessible and easy to clean bathing facilities, prioritisation of households with a PWI when rolling out water supply systems (particularly subsidies to support on-premises connections), and the development of simple lifting aids to support caregivers with the movement of PWI.

- Service-based interventions: This may include a range of health, social and economic support services targeted to either the PWI or the caregiver. The design of such initiatives needs to start by developing more robust processes for identifying people with incontinence in LMICs and their caregivers. This may require the refinement and validation of existing incontinence questionnaires so that these tools are suited to a broad range of LMICs (15). Programmes should also prioritise connecting PWI and their caregivers to each other and supporting the formation of DPOs related to incontinence. This way experiences can be shared and PWI and caregivers can advise programme implementers on appropriate contextualised incontinence management options.
- Health practitioner-targeted interventions: Ministries of Health should be responsible for identifying local language terms for incontinence and developing training on incontinence for all levels of health care professionals. Health care professionals should also be encouraged to actively ask about incontinence with patients who may be more likely to experience the condition. Basic training on incontinence may need to extend to people working within the private or informal sectors (such as private pharmacies or market vendors) if they are in a position to sell incontinence products or give advice on the condition.
- Community-level interventions: There is a need to normalise and raise awareness about incontinence and effective local coping strategies. Such initiatives should involve PWI and caregivers to ensure that this is communicated in an acceptable manner.

Limitations

This study was not able to include any older people with incontinence given that potentially eligible individuals were not willing to participate or acknowledge their condition. This is similar to findings of other studies which indicate that accepting incontinence in older age can be difficult and is an important first step in effective management (31,32). This study also purposely selected people with severe incontinence and excluded people with incontinence that could be rectified though medical or surgical intervention. However, there may be a great deal to learn from people with milder incontinence and this may be a much larger proportion of the population. While this study provides insight into the barriers and management of incontinence in Sindh Province, it is also possible that some of the more culturally specific practices are not generalizable to other LMIC.

Conclusion

Consistent with prior research our study highlights that that incontinence is a condition with complex health, psychological, social, economic, and cultural implications. These consequences affected not only the daily lives of PWI, but to an almost equal extent, it affected the lives of their caregivers. While some of these challenges related to incontinence management have been acknowledged previously, they appear to be exacerbated in LMICs due a lack of recognition of the condition and the absence of interventions by government or NGOs. Making incontinence management easier in LMIC settings needs to start by strengthening government systems to identify and form support networks for PWI and their caregivers. Appropriate solutions need to be developed in partnership with PWI and caregivers and contextualised not just by country, but potentially at a household level to meet individual needs and preferences. Incontinence management initiatives need to be designed for durability, affordability and sustainability and additional research is needed to document and evaluate feasible initiatives to support PWI and caregivers in LMICs.

References

1. Ali H, Lakhani N, Medical NS-TP, 2013 undefined. Urinary incontinence three months after delivery. theprofesional.com [Internet]. [cited 2021 Nov 11]; Available from: http://www.theprofesional.com/index.php/tpmj/article/download/1095/848

2. Gastroenterology PMJ-, 2004 undefined. Economic and personal impact of fecal and urinary incontinence. Elsevier [Internet]. [cited 2021 Nov 11]; Available from: https://www.sciencedirect.com/science/article/pii/S0016508503017244

3. Water HOWCAN, Better HA, The A, Of N, People V, Countries MI. Hygiene needs of incontinence sufferers FROM URINE AND / OR FAECAL INCONTINENCE IN LOW AND. 2015;(September).

4. Minassian VA, Drutz HP, Al-Badr A. Urinary incontinence as a worldwide problem. Int J Gynecol Obstet. 2003 Sep 1;82(3):327–38.

5. Ng K, Sivakumaran Y, … NN-D of the C, 2015 undefined. Fecal incontinence: community prevalence and associated factors—a systematic review. ingentaconnect.com [Internet]. [cited 2021 Nov 11]; Available from: https://www.ingentaconnect.com/content/wk/dcr/2015/00000058/00000012/art00026

6. Milsom I, Coyne K, Nicholson S, Kvasz M, urology CC-E, 2014 undefined. Global prevalence and economic burden of urgency urinary incontinence: a systematic review. Elsevier [Internet]. 2013 [cited 2021 Nov 11]; Available from: https://www.sciencedirect.com/science/article/pii/S0302283813008592

7. Sutherland S. The psychology of incontinence . Acad Press. 1976;

8. Grimby A, Milsom I, Molander U, … IW-A and, 1993 undefined. The influence of urinary incontinence on the quality of life of elderly women. academic.oup.com [Internet]. [cited 2021 Nov 11]; Available from: https://academic.oup.com/ageing/article-abstract/22/2/82/24798

9. Taylor D, Cahill J, Res DR-PH, 2014 undefined. Denial, shame and acceptance: generating base-line knowledge and understanding of fecal incontinence amongst long-term care residents and care providers. cameroninstitute.com [Internet]. 2014 [cited 2021 Nov 11];4(1):13–8. Available from: http://www.cameroninstitute.com/wp-content/uploads/2014/11/10.5923.j.phr_.20140401.03.pdf

10. Perera J, Kirthinanda DS, Wijeratne S, Wickramarachchi TK. Descriptive cross sectional study on prevalence, perceptions, predisposing factors and health seeking behaviour of women with stress urinary incontinence. BMC Womens Health. 2014 Jul 2;14(1).

11. Whitehead W, … OP-NC medical, 2016 undefined. Treating fecal incontinence: an unmet need in primary care medicine. ncmedicaljournal.com [Internet]. [cited 2021 Nov 11]; Available from: https://www.ncmedicaljournal.com/content/77/3/211.short

12. Link B, Lancet JP-T, 2006 undefined. Stigma and its public health implications. researchgate.net [Internet]. [cited 2021 Nov 11]; Available from: https://www.researchgate.net/profile/Andrew-Hargreaves/post/Where-can-I-get-good-scholarly-writings-on-social-inclusion/attachment/5cbc52ff3843b01b9b9b8407/AS%3A750086170619904%401555845887103/download/LINK+and+Phelan+2006+stigma+and+its+public+health+implications.pdf

13. Wang C, Li J, Wan X, Wang X, Kane RL, Wang K. Effects of stigma on C hinese women's attitudes towards seeking treatment for urinary incontinence. Wiley Online Libr [Internet]. 2014 Apr 1 [cited 2021 Nov 11];24(7–8):1112–21. Available from: https://onlinelibrary.wiley.com/doi/abs/10.1111/jocn.12729?casa_token=UIm3-frYO5QAAAAA:L3yQkw8YMmHCIN19sHwS8UOHqzPjwOgQRzC-mluzTzlvqnZpbTGrBmR7EYxTnWuNWek9GPj7MEayTGM

14. Hu T, Wagner T, Bentkover J, Leblanc K, Urology SZ-, 2004 undefined. Costs of urinary incontinence and overactive bladder in the United States: a comparative study. Elsevier [Internet]. [cited 2021 Nov 11]; Available from: https://www.sciencedirect.com/science/article/pii/S0090429503011774

15. Wilbur J, Morrison C, Bambery L, … JT-TLR, 2021 undefined. "I'm scared to talk about it": exploring experiences of incontinence for people with and without disabilities in Vanuatu, using mixed methods. Elsevier [Internet]. [cited 2021 Nov 11]; Available from: https://www.sciencedirect.com/science/article/pii/S2666606521001462

16. Organization WH. World report on disability 2011 [Internet]. 2011 [cited 2021 Nov 11]. Available from: https://apps.who.int/iris/bitstream/handle/10665/44575/?sequence=4

17. Biswas A, Development KSWR, 2008 undefined. Achieving Water Security for Asia: Asian water development outlook, 2007. Taylor Fr [Internet]. 2008 Mar [cited 2021 Nov 11];24(1):145–76. Available from: https://www.tandfonline.com/doi/abs/10.1080/07900620701760556

18. White S, Kuper H, Itimu-Phiri A, Holm R, Biran A. A qualitative study of barriers to accessing water, sanitation and hygiene for disabled people in Malawi. PLoS One. 2016 May 1;11(5).

19. Braun V, psychology VC-Q research in, 2006 undefined. Using thematic analysis in psychology. Taylor Fr [Internet]. 2006 [cited 2021 Nov 11];3(2):77–101. Available from: https://www.tandfonline.com/doi/abs/10.1191/1478088706QP063OA

20. Saldaña J. The coding manual for qualitative researchers [Internet]. 2021 [cited 2021 Nov 11]. Available from: https://books.google.com/books?hl=en&lr=&id=RwcVEAAAQBAJ&oi=fnd&pg=PP1&dq=Saldaña+J.+The+coding+manual+for+qualitative+researchers:+Sage%3B+2015.&ots=ecbLfLQn-a&sig=LqiSGOcXggGbYQWSzqCg6hwxd0g

21. OrganizaWorld Health. Towards a common language for functioning, disability and health: ICF. Int Classif [Internet]. 2002;1149:1–22. Available from: http://www.who.int/classifications/icf/training/icfbeginnersguide.pdf

22. Van Houtven CH, Voils CI, Weinberger M. An organizing framework for informal caregiver interventions: detailing caregiving activities and caregiver and care recipient outcomes to optimize evaluation efforts. BMC Geriatr 2011 111 [Internet]. 2011 Nov 22 [cited 2021 Nov 11];11(1):1–18. Available from: https://bmcgeriatr.biomedcentral.com/articles/10.1186/1471-2318-11-77

23. Talley KMC, Davis NJ, Peden-McAlpine C, Martin CL, Weinfurter E V., Wyman JF. Navigating through incontinence: A qualitative systematic review and meta-aggregation of the experiences of family caregivers. Int J Nurs Stud. 2021 Nov 1;123:104062.

24. 2013) JE-NOP (through, 2002 undefined. The psychosocial impact of incontinence on older

people: a review. search.proquest.com [Internet]. [cited 2021 Nov 11]; Available from: https://search.proquest.com/openview/d2cd47fa3f0f6b1659ba95e1b12bc023/1?pq-origsite=gscholar&cbl=45202

25. Arkan G, Beser A, Ozturk V. Experiences Related to Urinary Incontinence of Stroke Patients: A Qualitative Descriptive Study. J Neurosci Nurs [Internet]. 2018 Feb 1 [cited 2021 Nov 11];50(1):42–7. Available from: https://journals.lww.com/jnnonline/Fulltext/2018/02000/Experiences_Related_to_Urinary_Incontinence_of.11.aspx

26. Fischer N, Church P, Lyons J, McPherson AC. A qualitative exploration of the experiences of children with spina bifida and their parents around incontinence and social participation. Child Care Health Dev [Internet]. 2015 Nov 1 [cited 2021 Nov 11];41(6):954–62. Available from: https://onlinelibrary.wiley.com/doi/full/10.1111/cch.12257

27. Hayder D, Schnepp W. Experiencing and managing urinary incontinence: A qualitative study. West J Nurs Res [Internet]. 2010 Aug 4 [cited 2021 Nov 11];32(4):480–96. Available from: https://journals.sagepub.com/doi/abs/10.1177/0193945909354903?casa_token=E2gFxD-LrJEAAAAA%3Alxd-1U0MPjzUa9HSWvH79ZphhIfqWZx_SuUFOsLcow8Us4CAH8p_Qbys28Zi3f4i5N_RZsZRHndP

28. Gallagher M, Cns RN, Pierce LL, Crrn RC. Caregivers' and Care Recipients' Perceptions of Dealing with Urinary Incontinence. Rehabil Nurs [Internet]. 2002 Jan 2 [cited 2021 Nov 11];27(1):25–31. Available from: https://onlinelibrary.wiley.com/doi/full/10.1002/j.2048-7940.2002.tb01976.x

29. Diagnosis and Management of Fecal Incontinence : Official journal of the American College of Gastroenterology | ACG [Internet]. [cited 2021 Nov 11]. Available from: https://journals.lww.com/ajg/Fulltext/2004/08000/Diagnosis_and_Management_of_Fecal_Incontinence.34.aspx

30. Goal 6: Clean Water and Sanitation - SDG Tracker [Internet]. [cited 2021 Nov 11]. Available from: https://sdg-tracker.org/water-and-sanitation

31. Santini S, Andersson G, Lamura G. Impact of incontinence on the quality of life of caregivers of older persons with incontinence: A qualitative study in four European countries. Arch Gerontol Geriatr. 2016 Mar 1;63:92–101.

32. Horrocks S, Somerset M, Stoddart H, Peters TJ. What prevents older people from seeking treatment for urinary incontinence? A qualitative exploration of barriers to the use of community continence services. Fam Pract [Internet]. 2004 Dec 1 [cited 2021 Nov 11];21(6):689–96. Available from: https://academic.oup.com/fampra/article/21/6/689/508768

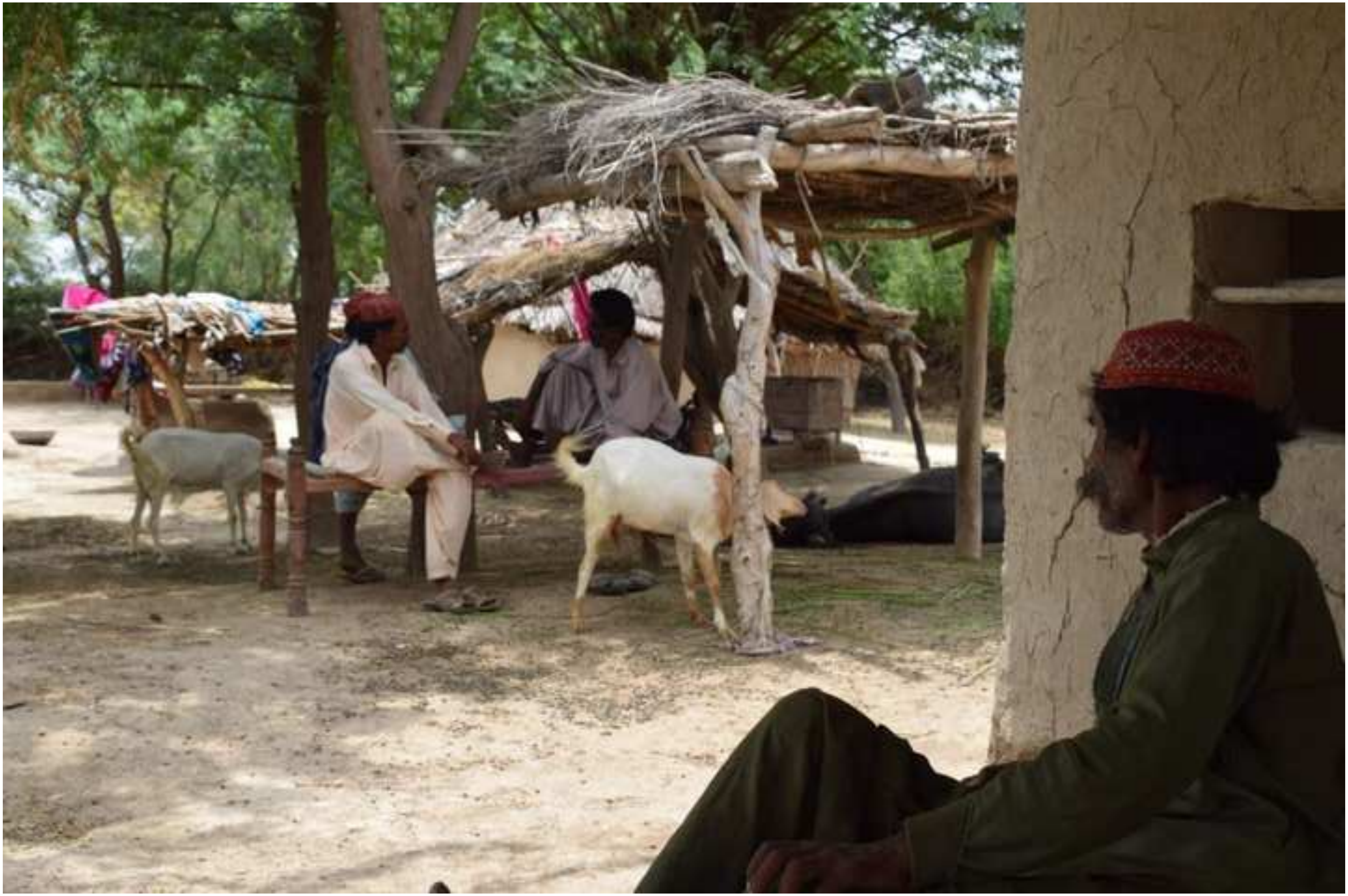

Click here to access/download;Figure;Figure 2.jpeg

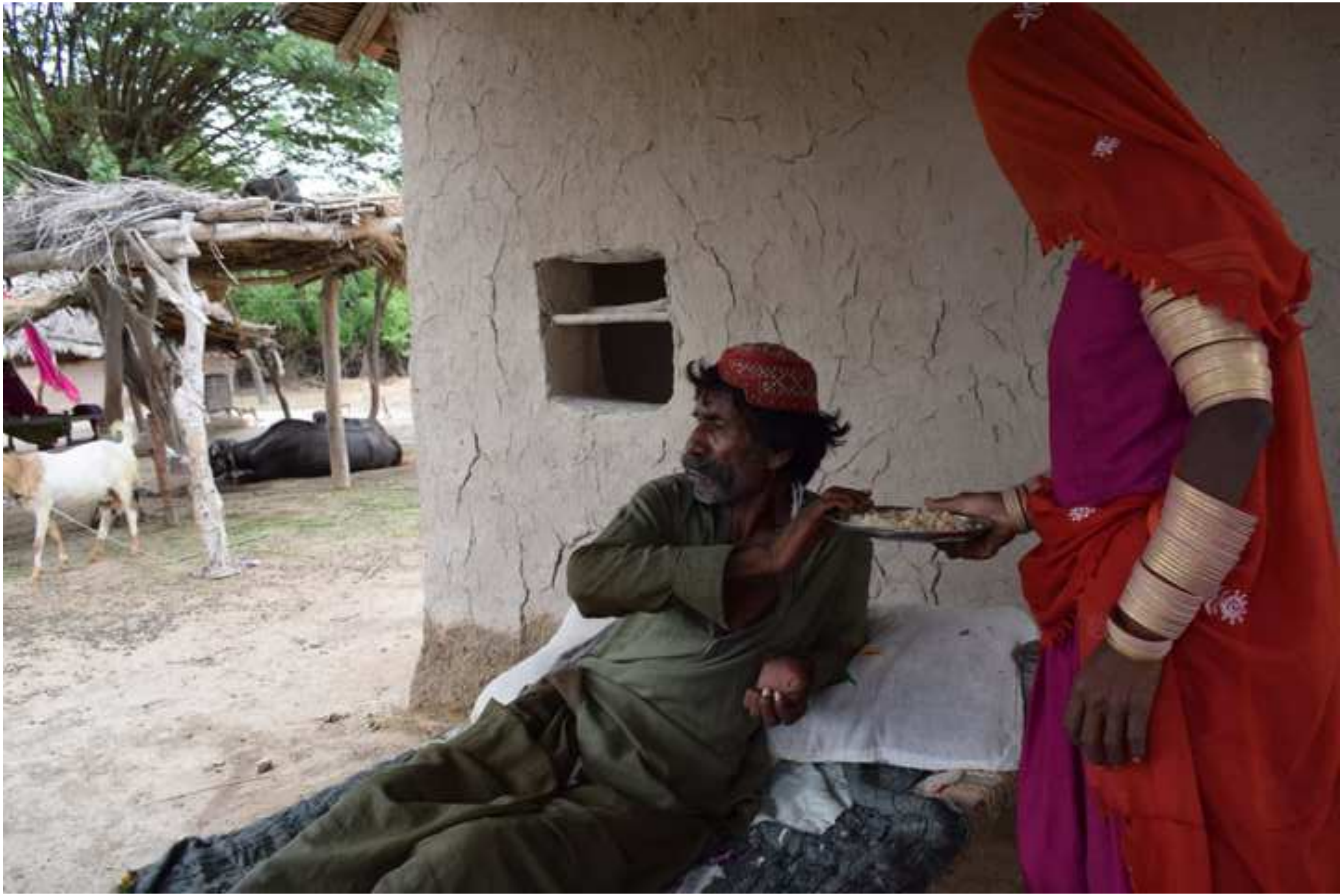

Click here to access/download;Figure;Figure 3.jpeg

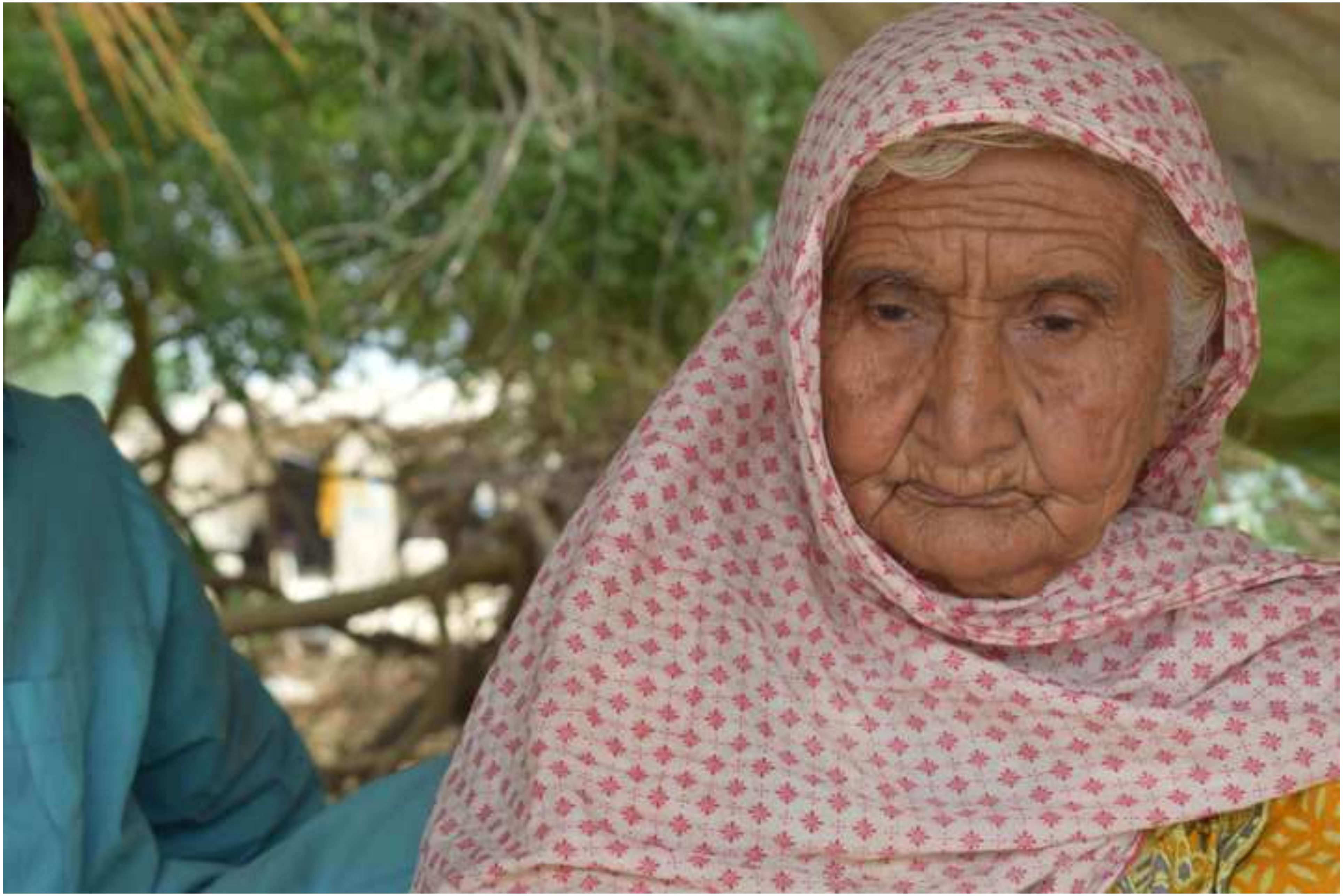

Click here to access/download;Figure;figure 4.png

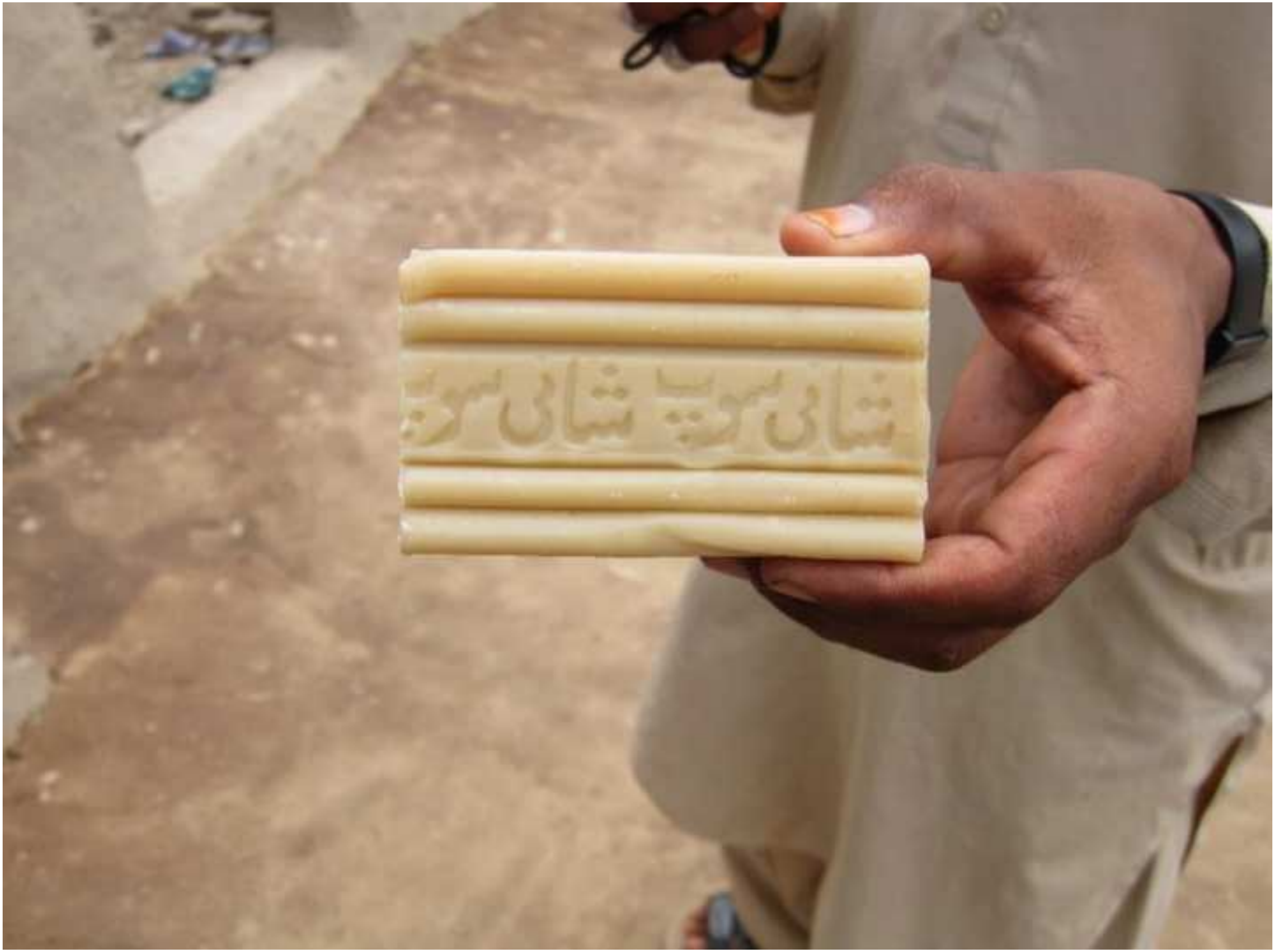

Click here to access/download;Figure;Figure 5 - ICF diagram.jpg

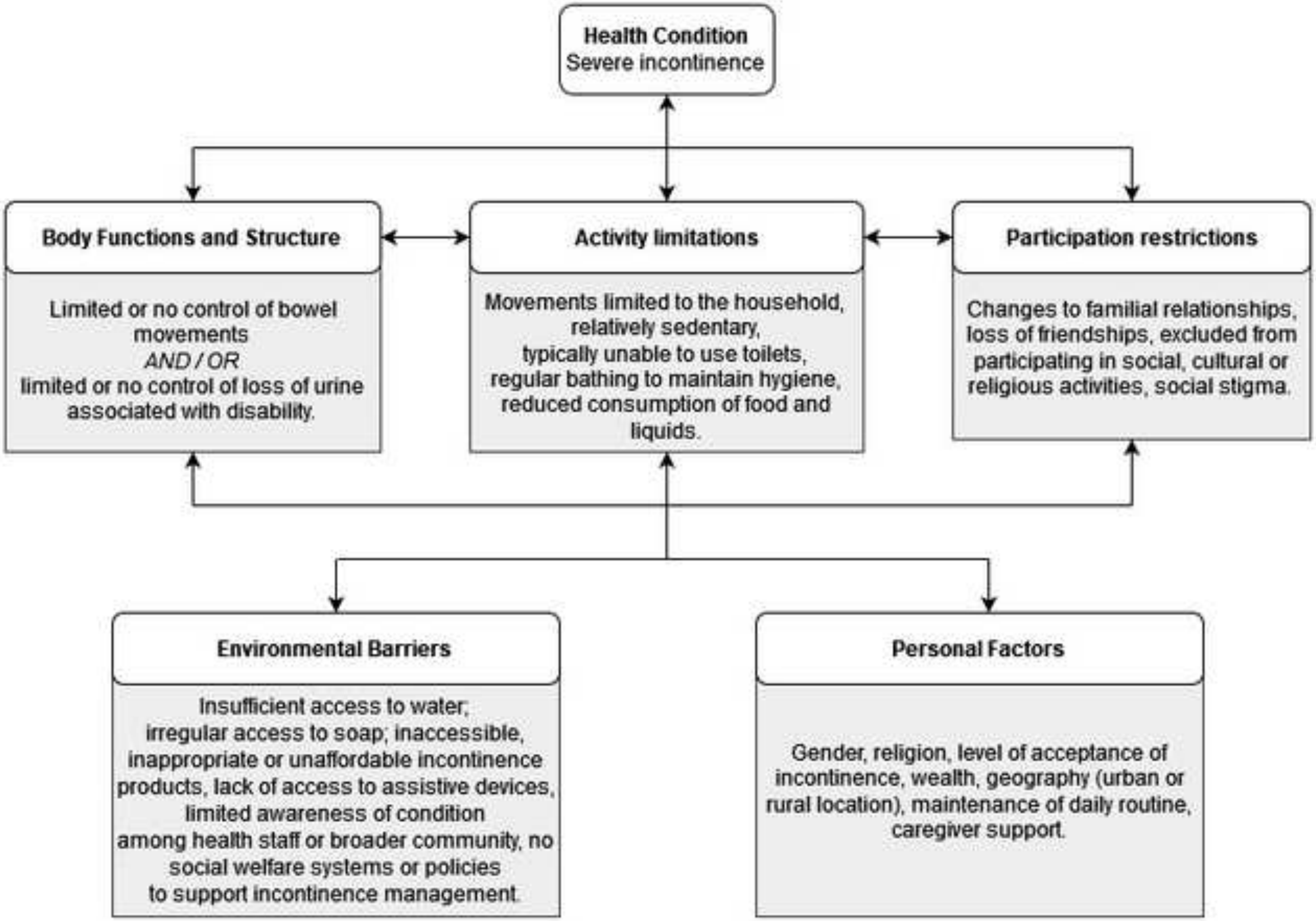

Click here to access/download;Figure;Figure 6 - CaregiverDiagram.jpg

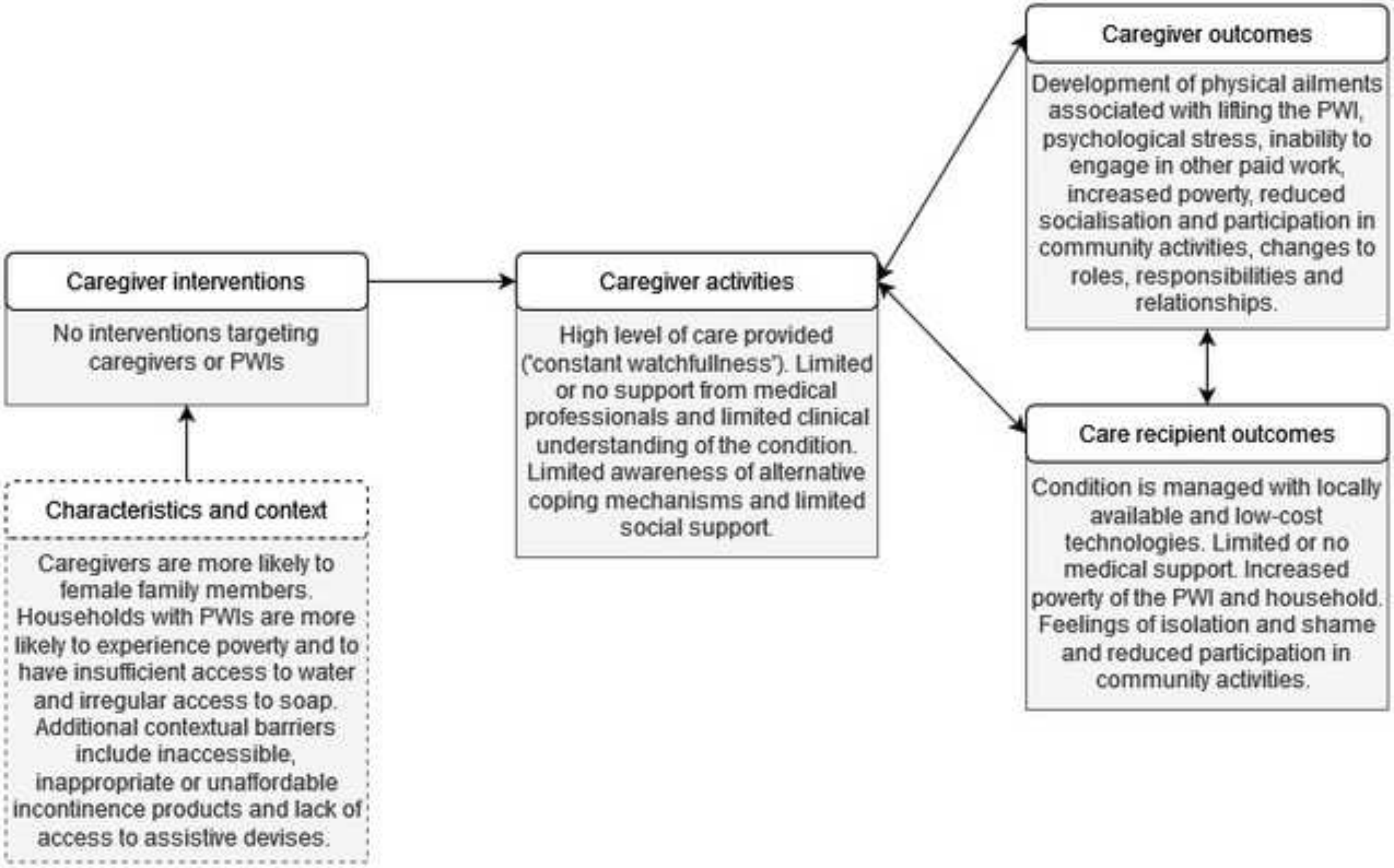

Supplement: Attachment — Submitted filename: PONE-D-22-04296_reviewer.pdf [file pone.0271617.s002.pdf]
