# Supplementary material for: Managing incontinence in low-and middle income-countries: A qualitative case study from Pakistan
Source: PLoS One. 2022 Jul 15;17(7):e0271617. doi: 10.1371/journal.pone.0271617 (PMC9286225; doi:10.1371/journal.pone.0271617)
Supplement: Attachment — Submitted filename: Review- Managing incontinence in LMICs.docx [file pone.0271617.s001.docx]

A valuable study and contribution to the emerging field of incontinence in LMICs and an enjoyable read. The Photovoice aspect was very powerful and of great interest to me. I have a few minor recommendations.

In the introduction to the paper, I would add a brief line about the fact that incontinence can affect anyone at any life stage. A good reference would be the Frontiers of Sanitation on incontinence produced by the Sanitation Learning Hub. This would give context to that fact that whilst this is a study on older people, anyone can be affected.

The section about the study site appears to read as though it is about Pakistan as a whole rather than Sindh province. It would be good to see a little more detail about the locality in general and its WASH context to paint a better picture of the conditions the people with incontinence are living in.

The methodology could be strengthened with a little more detail. Were there any challenges faced in the data collection process? Incontinence is a taboo issue and there can be ambiguity over definitions. How was incontinence defined in Urdu, and were there any issues in getting people to talk about it? A few words about the 6 step analysis method would help the reader to easily follow through. Was there any particular apparent reason why the older people without impairment refused to participate in the study?

On page 8, line one 163, it may be clearer to replace the word ‘predicted’ with ‘planned around’

The section on not being clean for God is really powerful. Interesting to see the religious diversity in this context. How were the minority Hindu families accessed and were there any more details on why they felt they needed to keep clean in the context of their religion? Are there geographical factors which made it easier to access them?

The section on use of local products for incontinence management jumps straight into findings from rural areas. Were there any patterns or distinct differences between urban and rural which can be outlined at the start as an overall pattern?

The five domains of interventions may read easier in the form of a simple and small table with one column for the type of intervention and a second column for description. This is very important information that should stand out. The recommendations would also be strengthened further by adding a little clarity on which actors should be responsible for implementing these.
