# Supplementary material for: Managing incontinence in low-and middle income-countries: A qualitative case study from Pakistan
Source: PLoS One. 2022 Jul 15;17(7):e0271617. doi: 10.1371/journal.pone.0271617 (PMC9286225; doi:10.1371/journal.pone.0271617)
Supplement: Attachment — Submitted filename: Responses to reviewers.docx [file pone.0271617.s003.docx]

**Responses to the editor:**

1. Please ensure that your manuscript meets PLOS ONE's style requirements, including those for file naming. The PLOS ONE style templates can be found at
   [https://journals.plos.org/plosone/s/file?id=wjVg/PLOSOne_formatting_sample_main_body.pdf](https://protect-eu.mimecast.com/s/bh3NCzXkji4q6M4sgkrC4?domain=journals.plos.org) and 
   [https://journals.plos.org/plosone/s/file?id=ba62/PLOSOne_formatting_sample_title_authors_affiliations.pdf](https://protect-eu.mimecast.com/s/9bNmCA1pkcEO4NEcYE3Lr?domain=journals.plos.org)

*Response: We have gone through this guidance and the manuscript now fully adheres to these guidelines. In particular we have adjusted the naming of all figures.*

2. We note that Figures 1 to 3 includes an image of a [patient / participant / in the study].
   As per the PLOS ONE policy ([http://journals.plos.org/plosone/s/submission-guidelines#loc-human-subjects-research](http://protect-eu.mimecast.com/s/3NqwCB6q0U8Kj78FWN6DK?domain=journals.plos.org)) on papers that include identifying, or potentially identifying, information, the individual(s) or parent(s)/guardian(s) must be informed of the terms of the PLOS open-access (CC-BY) license and provide specific permission for publication of these details under the terms of this license. Please download the Consent Form for Publication in a PLOS Journal([http://journals.plos.org/plosone/s/file?id=8ce6/plos-consent-form-english.pdf](http://protect-eu.mimecast.com/s/AVH7CD8vnC3Aq53Ik8GO8?domain=journals.plos.org)). The signed consent form should not be submitted with the manuscript, but should be securely filed in the individual's case notes. Please amend the methods section and ethics statement of the manuscript to explicitly state that the patient/participant has provided consent for publication: “The individual in this manuscript has given written informed consent (as outlined in PLOS consent form) to publish these case details”. If you are unable to obtain consent from the subject of the photograph, you will need to remove the figure and any other textual identifying information or case descriptions for this individual.

*Response: Given that the field-based data collection associated with this work was undertaken in 2017 we have been unable to reach the participants via their prior phone numbers to seek this additional permission. Our initial consent process was very through, and each participant featured in the photographs gave express permission for these to be used in publications related to this research, understanding that this would not be anonymous. However this consent process did not expressly state that we intended to publish in PlosOne and nor did we use your standard template for this. On this basis our only option was to remove all of these images which we feel is a great determined to the paper and is not in keeping with the wishes of the participants. However we have tried to still refer to these images and their captions so this is not fully lost. We have also left in the last image as this does not include any identifying information. For your reference we are including the consent form used for this research which was agreed to by all participants. We are including this for your review only and do not intend for this to be included as part of the manuscript. If there is any alternative to removing these images we would be keen to explore that.*

3. Please review your reference list to ensure that it is complete and correct. If you have cited papers that have been retracted, please include the rationale for doing so in the manuscript text, or remove these references and replace them with relevant current references. Any changes to the reference list should be mentioned in the rebuttal letter that accompanies your revised manuscript. If you need to cite a retracted article, indicate the article’s retracted status in the References list and also include a citation and full reference for the retraction notice.

*Response: We are not aware of any of the papers that we referenced being redacted. As per the feedback from Reviewer 2 we have amended some references.*

**Responses to Reviewers:**

Responses to Reviewer #1:

4. A valuable study and contribution to the emerging field of incontinence in LMICs and an enjoyable read. The Photovoice aspect was very powerful and of great interest to me. I have a few minor recommendations. In the introduction to the paper, I would add a brief line about the fact that incontinence can affect anyone at any life stage. A good reference would be the Frontiers of Sanitation on incontinence produced by the Sanitation Learning Hub. This would give context to that fact that whilst this is a study on older people, anyone can be affected.

*Response: Thank you for your kind comments. In the first paragraph we have added that incontinence can affect people across the life cycle. We have also included the reference you suggest.*

5. The section about the study site appears to read as though it is about Pakistan as a whole rather than Sindh province. It would be good to see a little more detail about the locality in general and its WASH context to paint a better picture of the conditions the people with incontinence are living in.

   *Response: We have edited this section to include more information on the local conditions in Sindh province.*
6. The methodology could be strengthened with a little more detail. Were there any challenges faced in the data collection process? Incontinence is a taboo issue and there can be ambiguity over definitions. How was incontinence defined in Urdu, and were there any issues in getting people to talk about it? A few words about the 6 step analysis method would help the reader to easily follow through.

*Response:* *We have described how there is no word in Urdu for incontinence and how we went about developing a definition that was used in the study. We have added extra detail to the analysis process.*

7. Was there any particular apparent reason why the older people without impairment refused to participate in the study?

*Response: The older people that were approached stated that they did not have incontinence, even though their caregivers confirmed it. We have not added anything to the text here as this was already stated.*

8. On page 8, line on*e* 163, it may be clearer to replace the word ‘predicted’ with ‘planned around’

*Response: This has been changed in the text.*

9. The section on not being clean for God is really powerful. Interesting to see the religious diversity in this context. How were the minority Hindu families accessed and were there any more details on why they felt they needed to keep clean in the context of their religion? Are there geographical factors which made it easier to access them?

*Response: There are certain districts in Sindh that have a higher number of Hindus living in them and those districts were part of the study. We did not purposively sample on the basis of religion so we did not know they were Hindu when we approached them to be part of the study. This detail came out through the interviews. As described in the text through their attitudes towards incontinence because of their religion seemed consistent with Muslim participants.*

10. The section on use of local products for incontinence management jumps straight into findings from rural areas. Were there any patterns or distinct differences between urban and rural which can be outlined at the start as an overall pattern?

*Response: Urban participants did not bring up using any unique alternatives (such as the use of straw or sand) for incontinence management. They relied more on water and soap. This has been clarified in the text of the manuscript.*

11. The five domains of interventions may read easier in the form of a simple and small table with one column for the type of intervention and a second column for description. This is very important information that should stand out. The recommendations would also be strengthened further by adding a little clarity on which actors should be responsible for implementing these.

*Response: We have taken the reviewers advice and transformed this into a table (Table 4)*

**Responses to Reviewer #2:**

12. This is a well-written and concise account of a very interesting study. I saw the results presented at a conference several years ago and have been looking forward to its publication! I have no overarching comments, I think it's great. I have a few small comments on the manuscript, mostly grammatical, but of relevance to the authors a couple of places where I think the citation might be incorrect/a couple of other citations could be added to assist the reader to learn more.

*Response: These comments have been incorporated throughout the paper.*
